# Radiolabelling and bioequivalence of modified Tamoxifen solid lipid nanoparticles as a targeted chemotherapeutic drug

**DOI:** 10.1007/s13346-025-01865-1

**Published:** 2025-05-07

**Authors:** Rania S. Abdel-Rashid, Eman S. El-leithy, Ismail T. Ibrahim, Khaled M. Attallah

**Affiliations:** 1 Department of Pharmaceutics and Industrial Pharmacy, Faculty of Pharmacy, Helwan University, Ain Helwan, P.O. Box 11795, Cairo, Egypt; 2 Nanotechnology Research Center, Helwan University, Cairo, Egypt; 3 Labeled Compound Department, Hot Lab Center, Egyptian Atomic Energy Authority, P.O. Box 13759, Cairo, Egypt

**Keywords:** Cancer, Tamoxifen, Solid lipid nanoparticles, Technetium-99m labelling, Biodistribution

## Abstract

There are several types of breast cancer where the breast's cells proliferate uncontrollably. A selective oestrogen receptor modulator called Tamoxifen citrate (TAM) is used to treat and prevent breast cancer in both men and women. TAM is classified as class II under the biopharmaceutical categorization system (BCS) of medications. It exhibits low plasma levels, which can result in therapeutic failure due to its poor water solubility. To improve its chemotherapeutic efficiency and drug targeting, nanotechnology was exploited. In this article, TAM-loaded SLNs were prepared, characterized, and radiolabelled with Technetium-99m ([^99m^Tc]Tc) using stannous salts followed by the assessment of their radiochemical efficiency and in vivo biodistribution compared to the radiolabelled free TAM ([^99m^Tc]Tc-TAM). The results showed that the concentration of lipid had a highly prominent effect on the particle size and encapsulation efficiency of the drug, where the best selected formula showed spherical, non-aggregated morphology with a 134.6 ± 0.3 nm size and 83.9 ± 2.5% drug encapsulation. The radiolabelling purity was more than 97.4%, and it was stable for at least 6 h. In solid tumor-bearing mice, [^99m^Tc]Tc-TAM-SLNs exhibited around 3 times more uptake than [^99m^Tc]Tc-TAM solution. Accordingly, [^99m^Tc]Tc-TAM-SLNs can be suggested as a useful targeted delivery strategy for chemotherapy drugs.

**Graphical Abstract:**

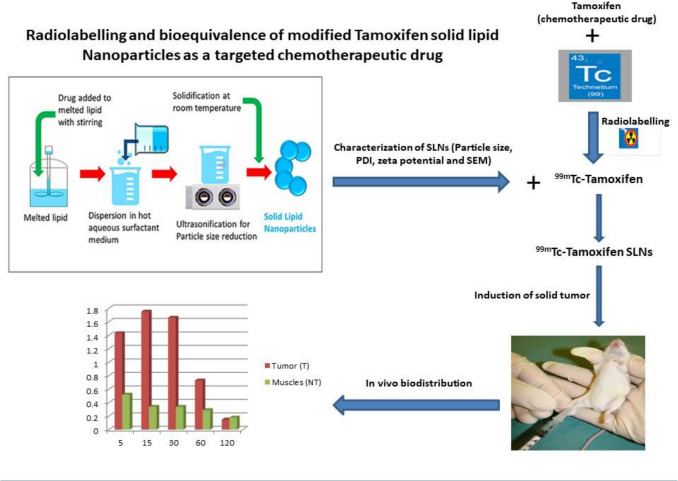

## Introduction

Breast cancer is the uncontrollable growth and reproduction of breast cells, initiating from the lobules or ducts in the breast with high exposure to show metastatic behavior reaching other areas in the patient’s body [1]. Stopping metastasis could be efficiently achieved in breast cancer using different chemotherapeutic pathways via hormone therapy or immunotherapy and cytotoxic medications [2]. When developing chemotherapeutic drugs, the therapeutic efficacy, effect on patient’s lifestyle, and the possible toxicity are important considerations [3]. Hence, there is tremendous efforts on reforming the traditional cancer treatments reaching high therapeutic efficacy and safety profile by using particulate drug carrier systems [4].

Recently, nanotechnology has emerged as one of the most promising approaches applied for the improvement of cancer treatment efficacy and prognosis [5]. Liposomes and polymeric nanoparticles are examples of powerful cytotoxic drug delivery nanotechnologies; however they have shown a number of problems concerning physical stability, controlled release mechanisms and preservation of labile medicines from degradation [6]. These limitations are generally absent in the case of SLNs [7]. The drug delivery to solid tumors is a big challenge, as most anti-cancer medications have a wide volume of distribution after injection which limits their therapeutic index due to their high level of toxicity in healthy tissues. Encasing these drugs in a drug delivery system such as SLNs efficiently reduces the volume of distribution while increasing the drug concentration in the tumor site [8]. The SLNs are an intriguing colloidal drug delivery system made of lipids that are solid at room temperature. They are distinguished by their high physical stability, ease of manufacture, capacity to prevent the degradation of labile medications, biocompatibility, and biodegradability [9, 10].

SLNs encounter several challenges during preclinical development, much like other nano-systems. The FDA identified seven priority topics, three of which stressed the need for researching the pharmacokinetics of treatments based on nanotechnology for patients [11]. The priorities include determination of nanoparticle biodistribution in different tissue types after systemic administration and the creation of imaging techniques to monitor the long-term trajectory of nanoparticles in animal systems [12]. Subsequently, in vivo biodistribution is an important factor to be considered when designing and testing novel nanoparticulate systems. It could also be a very good indicator for selectivity and target ability of the nanoparticulate systems and a credible means for their further development [6]. Due to its ideal imaging characteristics, including its 140 keV emission and short half-life of 6.0 h, technetium-99m ([^99m^Tc]Tc) is the most extensively used radionuclide. These features enable it to be employed as an essential tool for the diagnosis and treatment of numerous illnesses or dysfunctions of human body systems. Furthermore, ^99m^Tc is a perfect radionuclide for radiolabelling medications and nanoparticles due to its imaging properties and cost-effectiveness [13, 14].

Radiocolloids of technetium- 99m prepared by using reduced sodium pertechnetate (Na[^99m^Tc]TcO_4_) demonstrated high sensitivity for radiolabelling of nanoparticles. To achieve the highest labelling efficiency, the reaction mixture's pH and stannous salt content must be optimized [15, 16].

TAM is a non-steroidal potent oestrogen antagonist with partial agonist properties. It is the recommended treatment for all stages of oestrogen receptor-positive breast cancer, as well as for long-term preventive treatment in postmenopausal and high-risk women [17]. Hot flashes (64%), mood swings (6%), depression, irritability or vaginal dryness (35%), weight gain (6%), and sleep issues (36%) are among the side effects of Tamoxifen. Patients are more susceptible to oxidative stress-mediated hepatotoxicity when they receive prolonged treatment and accumulate doses. It is advised that women with hormone receptor-positive breast cancer take it continuously for five years to reduce the risk of invasive breast cancer transformation and recurrence. Taking TAM for ten years may be even more beneficial, according to the most updated therapeutic regimen [18, 19]. TAM has been investigated for its feasibility to be encapsulated in SLNs and has demonstrated promising results for efficient delivery with suitable drug loading [7]. It was also reported that free TAM could be successfully radiolabeled with TC- 99 to explore the role of Tc-99m-tamoxifen in Estrogen Receptor-expressing lesions in breast cancer patients [20].

For the sake of exploiting the previous data, this work's objective was to look at the potential of using SLNs as a delivery mechanism to enhance the therapeutic objectives and decrease unfavorable side effects of TAM by tracing the in vivo distribution of prepared radiolabeled [^99m^Tc]Tc-TAM-SLNs. First, the evaluation of how the formulation variables affected the zeta potential, particle size, and entrapment efficiency was studied. Furthermore, [^99m^Tc]Tc-TAM complex was incorporated into SLNs to investigate their biodistribution in mice bearing solid tumor.

## Materials and methods

### Materials

Amriya Pharmaceutical Co. (Alexandria, Egypt) generously supplied TAM and soybean lecithin (Phospholipon 90G, 90% w/w of phosphatidyl choline). United Company for Chemical and Medical Preparations (Cairo, Egypt) supplied the 99% methyl alcohol (absolute). Stearic acid and tween 80 were purchased from EL-Gomhoria Company, Cairo, Egypt. Chloroform, Labscan Ltd, Dublin, Ireland. We bought cellulose membrane for dialysis tubing (Molecular Weight Cut Off 12,000–14,000) from Sigma Aldrich in Germany. The [^99^Mo]Mo/[^99 m^Tc]Tc generator provided by the Egyptian Second Research Reactor (ETRR- 2) of the Radioisotopes Production Facility (RPF), Egyptian Atomic Energy Authority, was used to elute sodium pertechnetate (Na[^99m^Tc]TcO_4_). All other chemicals and solvents were of analytical grade.

### Methods

#### Preparation of TAM-SLNs

The SLNs were made using a modified low-temperature solidification and melt emulsion/ultrasonication process [21, 22]. Weighed 20 mg of TAM were dissolved in methanol and poured into different concentrations of lipid melt (stearic acid) at 65–70 °C to produce a uniform, transparent drug-lipid combination. A crude emulsion was produced by emulsifying the drug-lipid mixture in an aqueous solution of a surfactant (Tween 80 or lecithin), which was mechanically stirred at 6,000 rpm and heated to 10 °C above the melting point of the lipids. Then, using an ultrasonic processor, the crude emulsion was ultrasonically treated in a water bath for 10 min at 90 °C [23]. After cooling to room temperature, the SLN nanoparticles were produced and suspended in the medium. The effects of surfactant type, concentration, and lipid concentration were studied (Table 1).

**Table 1:** Composition of different prepared TAM-SLNs formulas

Formula code	Stearic acid conc (%w/v)	Tween 80 conc (%w/v)	Lecithin conc (%w/v)
SLN1	2	0.5	-
SLN2	3.5	0.2	-
SLN3	2	-	0.5
SLN4	3.5	-	0.2

### Characterization of prepared TAM-SLNs formulas

#### Drug loading and entrapment efficiency

A reverse phase column C18 phenomax 150 × 4.6 mm with a porosity of 5 × 10^–3^ mm as a stationary phase was used in HPLC with a Shimadzu chromatographic system to calculate the percentage of drug loading capacity (%DL) and entrapment efficiency (%EE). For Tamoxifen citrate separation, Monteagudo et al. (2012) [24] determined that a polar mobile phase of triethylamine acetate buffer: methanol with a ratio of 24.3:75.7 v/v was ideal. The drug was detected by UV at 275 nm, and the injection volume was 50 µL with a flow rate of 1.5 mL/min. The %DL and %EE refer to the percentage of drug entrapped in SLNs according to the following equations, respectively:

1 $${\%DL}=\frac{\text{Amount of drug entrapped in SLNs }}{\text{Total weight of lipids incorporated in SLNs}}\times 100$$

2 $${\%EE}=\frac{\text{Amount of drug actually present }}{\text{ Total amount of drug added}}\times 100$$

#### Assessment of particle size, PDI, and zeta potential

Using a Zetasizer (Malvern NanoZS, Malvern Instruments, Malvern, UK), the dynamic light scattering method was used to quantify the mean particle size (PS) and polydispersity index (PDI) of TAM-SLNs. Disposable cells were used to average five observations at a 173° angle in order to acquire the results. Disposable simple folded capillary zeta cells were used to measure the zeta potential (ZP) of the samples. At 25 ± 2 °C, every measurement was made at least five times.

#### Imaging with transmission electron microscope

Using a transmission electron microscope (Thermo Scientific Apreo S, Waltham, MA, USA) at high vacuum, the size and surface characteristics of nanoparticles were investigated. For this, a Leica EMACE 600 (Leica Microsystems, Wetzlar, Germany) brand coating apparatus was used to first coat the samples with 80% gold and 20% palladium at a thickness of 7 nm. The vacuum used to prepare the coating was 5 × 10^−4^ mbar. The coated samples were scanned under elevated voltage settings of 5 kV and at a magnification range of × 50,000.

#### Selection of formula candidate for further studies

Based on the results of previously performed investigations, a certain criterion was implemented for selection of the best formula to be subjected for further testing. The formula showing the least particle size, least PDI accompanied with maximum encapsulation efficiency and Zeta potential will be considered as the best produced preparation.

#### Radiolabelling process and quality control test

To prepare TAM solution; 10 of tamoxifen was dissolved in 1 ml methanol: H_2_O (2:1) to ensure proper solubility before radiolabelling. Technetium-99m is typically obtained as sodium pertechnetate (Na^99m^TcO₄) by using 0.9% saline solution from a molybdenum-99 generator,in a hepta-oxidation state, which cannot label any chemical compound through direct addition. So, shortly before the labelling operation, [^99m^Tc]Tc reduction is required to convert [^99 m^Tc]Tc + 7 from the hepta state to a beneficial lower oxidation state, which can combine with the ligand to generate the radiopharmaceuticals [25]. This reduction process was done by using SnCl_2_ as a reducing agent. The reduced technetium [^99m^Tc]Tc interacts with TAM using the direct labelling technique forming the radiolabeled complex. The radiolabelled SLNs were prepared using the radiolabelled TAM, following the same procedure described previously. In order to identify the ideal reaction conditions, the required amounts (50–5000 µg) of TAM were transferred into clean 10-mL vials which were kept under positive N_2_ pressure. Then, the pH was adjusted using different volumes of 0.1 M HCl, 0.1 M NaOH or buffer solutions. After that, the required amount of SnCl_2_·2H_2_O (10–200 µg) and then 100 µL of freshly eluted pertechnetate solution (195 MBq) were added to each vial at different time intervals.

Each point's experiments were carried out in triplicate, and the student's t-test was used to assess data differences. Mean ± SD is used to present the results. A significant threshold of P < 0.05 was established. For conclusive results, the radiochemical purity was assessed and optimized utilizing both thin-layer chromatography (TLC) and paper electrophoresis. Acetone was utilized as the developing solvent in thin-layer chromatography to determine the free ^99m^TcO_4_^−^, while ethanol, water, and ammonium hydroxide (2: 5: 1) was used as a developing solvent to calculate the (R/H)-[^99m^Tc]NaTcO_4_ [26, 27].

Thus, the contribution of [^99m^Tc]Tc-ligand can be calculated as follows:

3 $${\% }\left[{}^{99\text{m}}\text{Tc}\right]\text{Tc}-\text{ligand}=100-\left({\% Free }\;{}^{99\text{m}}{\text{TcO}}_{{4}^{-}}+{ \%}\left(\text{R}/\text{H}\right)-\left[{}^{99\text{m}}\text{Tc}\right]{\text{NaTcO}}_{4}\right)$$

The strips were dried, cut into 0.5 cm pieces, and counted in a well-type γ-scintillation counter once the mobile phase had fully developed. A Whatman paper sheet measuring 2 cm in width and 47 cm in length was used for the paper electrophoresis study. A reaction mixture containing 1–2 μL was deposited on the paper sheet 12 cm away from the cathode edge. Normal saline solution (0.9% w/v) was used as the electrolyte solution for the 1.5-h electrophoresis, which was conducted at a voltage of 300 V. Following full development, the paper was taken out, dried, and cut into 0.5 cm long strips, which were subsequently counted in a well-type γ-counter [28].

#### Determination of [^99m^Tc]Tc-TAM-SLNs and [^99m^Tc]Tc-TAM in vitro stability

For 24 h, the in vitro stability of both [^99m^Tc]Tc-TAM-SLNs and [^99m^Tc]Tc-TAM were assessed at room temperature (25 ± 1 °C). Additionally, they were evaluated at 37 °C with human serum (compound: serum ratio of 1:8). Samples (2 µL, n = 3) were extracted from the reaction mixture at the proper intervals (1, 2, 4, 6, and 24 h post-incubation), and were subjected to the previously described chromatographic procedure and the radiochemical purity was assayed using a well-type gamma counter (Sesa Uniscaller) [26].

#### In-vivo Biodistribution study

With ethical clearance number 17PA/23, all animal studies were performed in accordance with EAEA NCRRT-REC criteria, and the study was carried out in accordance with national and international laws controlling the use of radioactive material. For the ^99m^Tc-ligand complexes'in vivo biodistribution investigations, four groups of twelve female Swiss Albino mice each were used (25–30 gm). Each animal received a 0.2 ml injection in the tail vein of a freshly produced solution comprising 200–400 KBq of [^99m^Tc]Tc-TAM-SLNs. For the necessary amount of time, the mice were housed in metabolic cages. After administration, at appropriate time intervals of 15, 60, 120, and 240 min, mice were anesthetized and blood samples were withdrawn by cardiac puncture into pre-heparinized polypropylene tubes. Tissue samples from the main organs were isolated, washed with 0.9% saline, weighed in pre-weighed tubes, and their activity was measured in a well-type γ-scintillation counter (SR- 7) [29]. The radioactivity uptake in each tissue sample was calculated as a fraction of the injected dose per gram (ID/g). It was estimated that the weight of muscle, bone, and blood constituted 7%, 10%, and 40% of the total body weight, respectively [30].

A solid tumor was induced in Swiss Albino mice using Ehrlich ascites carcinoma cells (EAC), a model based on a mouse mammary carcinoma [31]. In female Swiss Albino mice, a line of Ehrlich ascites carcinoma (EAC) was maintained by intraperitoneal (I.P.) transplantation of 2.5 X 10^6^ tumor cells/mouse. Aseptic needle aspiration was used to extract EAC cells. 2.5 X 10^6^ cells were counted microscopically using a hemocytometer in 0.1 mL of the ascitic fluid after it was diluted with sterile saline. Then, 0.2 mL of the solution was injected intramuscularly in the right thigh muscle to create a solid tumor [32, 33].

#### Pharmacokinetic behavior

The WinNonlin program (Ver. 1.5, Scientific Consulting Inc., Cary, NC) was used to calculate the pharmacokinetics parameters of both the plain drug and the formulation using non-compartment analysis [34]. The mean amount of radioactivity uptake (%ID/g) in blood and solid tumor samples was plotted against time (h). The radioactivity uptake is a direct indication of the amount of labelled TAM (either in [^99m^Tc]Tc-TAM solution or loaded in the [^99m^Tc]Tc-TAM-SLNs). This relationship allows recording the maximum concentrations of their uptake (C_max_) and the time for maximum uptake (T_max_). Additionally, the area under the curve from 0 to 240 min (AUC _(0–240)_, min %ID/g), and from 0 to infinity (AUC _(0-∞)_, min %ID/g), were estimated. The capacity of the formula to target the tumor through the intravenous route has been determined by the drug targeting efficiency (DTE) and the relative targeting efficiency (RTE) [35]. The AUC used here is that from 0 to 240 min and from 0 to ∞ following intravenous administration for both drug and delivery system ([^99m^Tc]Tc-TAM solution and [^99m^Tc]Tc-TAM-SLNs) and the plain drug. DTE is defined as the average partitioning time ratio between the tumor and the blood for both the nanoformula and the plain drug and can be calculated by dividing the AUC from 0 to 240 min for the tumor and blood. The DTE and the RTE were calculated using the following formulas [36].

4 $${DTE\%}=\frac{AUC0-240\;tumor}{AUC0-240\;blood}{ X \%}$$

5 $$RTE\%=\frac{Organ\;AUC0-240\;{\;of\;TAM\;loaded\;SLNs}}{{Organ\;AUC}0-240\;{of\;free\;Tamoxifen}}{\times}100$$

## Results and discussion

### Drug loading and encapsulation efficiency of TAM in SLNs

The nanoparticles were successfully prepared with 90% yield, showing a homogenous milky suspension and drug encapsulation ranging from 71.2 ± 4.7 to 90.3 ± 6.3%. The formulas were tested for their physical and chemical characters. The obtained results, as shown in Table 2, demonstrated that a rise in concentration of stearic acid exhibited a considerable increase in the encapsulation efficiency, which would be attributable to increasing the viscosity of the dispersion system and low water solubility of the medication. The results illustrate that the percentage of drug loading and TAM encapsulation were not significantly affected by the addition of Tween 80 (SLN2) or lecithin (SLN3). The higher drug entrapment efficiency observed with stearic acid SLNs was attributed to the effect of long-chain fatty acids attached to triglycerides that resulted in increased accommodation of lipophilic drugs [22].

**Table 2:** The characterization of TAM-SLNs formulations

	%DL	%EE	PS (nm)	PDI	ZP(mv)
SLN1	6.2 ± 0.3	71.2 ± 4.7	122.4 ± 10.3	0.521 ± 0.3	− 25.1 ± 0.3
SLN2	7.1 ± 0.2	85.5 ± 5.8	188 ± 0.3	0.624 ± 0.3	− 30.4 ± 0.3
SLN3	5.9 ± 0.4	83.9 ± 2.5	134.6 ± 0.3	0.422 ± 0.3	− 31.2 ± 0.3
SLN4	7.1 ± 0.9	90.3 ± 6.3	202.5 ± 0.3	0.601 ± 0.3	− 36.8 ± 0.3

### Characterization of particle size, PDI and zeta potential

The PS, PDI, and ZP seemed to be affected by both the type and concentration of surfactant, where the phospholipid (lecithin) had some prominent effect in decreasing particle size and increasing ZP. As shown in Table 2. The increase in particle size in proportion with the increase of stearic acid concentration from 2% w/v to 3.5% w/v could be caused by the high melting point of stearic acid and its viscosity [37]. It was also reported that high lipid content may negatively affect the efficiency of the homogenization and may also lead to an increase of surface area due to an increase in the number of particles, which require higher amounts of surfactant to stabilize them [38].

### Scanning electron microscope (SEM)

**Fig. 1:**
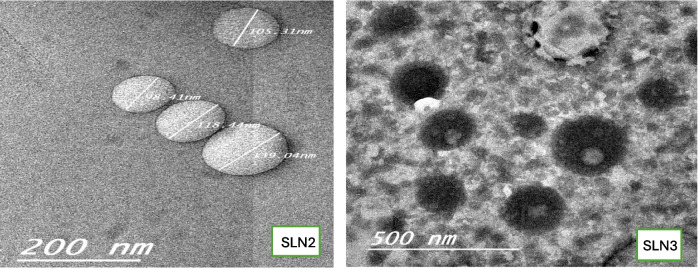
SEM images for TAM-SLNs formulas

The prepared SLNs showed a nearly spherical appearance with a dull color based on the absorption of the used dye by stearic acid. The surface was smooth, as shown in Fig. 1. The SLNs prepared using Tween 80 as a surfactant showed high aggregation and less darkness compared to the SLNs prepared in the presence of phospholipid (lecithin). The results agreed with the characteristic of lecithin, which favors a large oil/water interface providing an additional interfacial area, thus leading to smaller particle sizes and decreased particle aggregation [39].

### Selection of the best SLN formula

The formula coded SLN3 was selected according to the characterization results, where it showed 83.9 ± 2.5 drug entrapment and 134.6 ± 0.3 nm mean particle size with a PDI 0.422 ± 0.3, and ZP of − 31.2 ± 0.3 mv. The formula was chosen as the most reasonable candidate for the radiolabeling and the in vivo biodistribution study.

### Radiolabelling conditions

The radiolabelling technique of TAM was successful, as TAM was labelled with 97.4% purity. In case of TLC, the employed mobile phase (acetone) could identify the [^99m^Tc]Tc-ligand complex and reduced/hydrolyzed (R/H)-[^99m^Tc]NaTcO_4_ as they stayed at the origin with R_f_ = 0.0–0.1, while free pertechnetate was moved with the solvent front to R_f_ = 0.8–1.0. Using ethanol, water, and ammonium hydroxide (2: 5: 1) as a developing solvent to calculate the (R/H)-[^99m^Tc]NaTcO_4_, which stayed at the origin with R_f_ = 0.0–0.1, while other species moved with the solvent front (R_f_ = 0.8–1.0)[26].

**Fig. 2:**
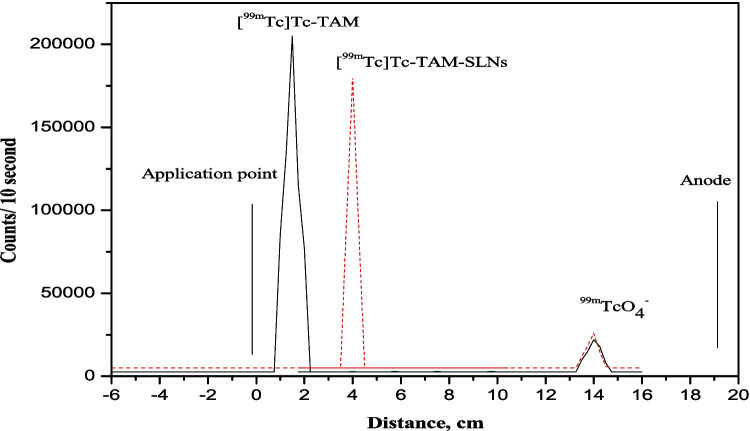
Electrophoresis radiochromatogram of [^99m^Tc]Tc-TAM and [^99m^Tc]Tc-TAM-SLNs

**Fig. 3:**
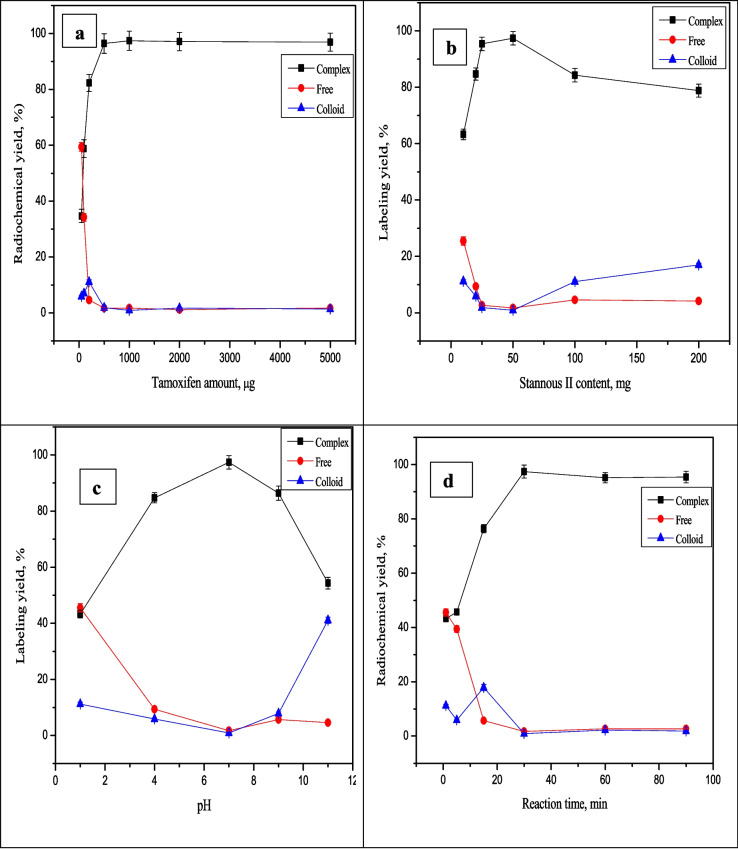
Variation of the radiochemical purity of [^99m^Tc]Tc-TAM as a function of substrate amount (**a**), Stannous II amount (**b**), pH (**c**) and reaction time (**d**)

Figure 2 illustrates the electrophoresis radiochromatogram of [^99m^Tc]Tc-TAM, [^99m^Tc]Tc-TAM-SLNs and free pertechnetate. The [^99m^Tc]Tc-TAM moves towards anode with R_f_ = 1.5–2, while the [^99m^Tc]Tc-TAM-SLNs moved with R_f_ = 4 and free pertechnetate moves towads anode with R_f_ = 14. Figure 3 provides a full explanation of the optimization parameters on the radiochemical purity, including the effects of substrate amount, reducing agent amount, pH, and reaction time.

As seen in Fig. 3(a), the percent labelling purity was 34.7% at low substrate amounts of TAM (0.05 mg). Because there was not enough substrate to form the complex with all of the reduced technetium, the labelling purity was low. The radiochemical purity increased to 97.4% of its maximum value when the ligand amount was increased to 1 mg. By increasing the ligand amount above the optimum value (1 mg), the labelling purity was nearly the same. At pH 7, the impact of stannous chloride content on the radiochemical purity percentage of [^99m^Tc]Tc-TAM was investigated. The impact of Sn (II) content on the percent labelling purity of [^99m^Tc]Tc-TAM-SLNs is displayed in Fig. 3(b). 97.4% was the efficient labelling purity attained with the use of the optimal amount of Sn (II), which was 50 µg.

The presence of a large amount of free pertechnetate at a concentration of 10 µg of Sn (II) can be explained by the fact this amount of tin chloride was not sufficient to completely reduce all the pertechnetate to form the [^99m^Tc]Tc-complex. The amount of colloid increased, and the purity of the labelling was decreased with increasing amounts of SnCl_2_.2H_2_O. The creation of ^99m^Tc-Sn-colloid may have been caused by the enhanced hydrolysis of Sn (II), which produced Sn colloids, and these species can compete with the ligand for the reduced technetium- 99m. It is evident from Fig. 3(c) that the labelling purity is influenced by the reaction mixture's pH. The maximum labelling purity of the preparation was obtained at pH 7 and was equal to 97.4% with a low percentage of free pertechnetate and [^99m^Tc]Tc-Sn-colloid. The primary radiochemical impurity, %(R/H)-[^99m^Tc]NaTcO4, which was 41.1% at pH 11, formed at pH values below or above the ideal pH, reducing the radiochemical output. Figure 3(d) demonstrated that a higher labelling purity was correlated with a longer reaction time. At 30 min of reaction time, the maximum purity of 97.4% was attained.

### In vitro stability of radiolabelled compounds

**Fig. 4:**
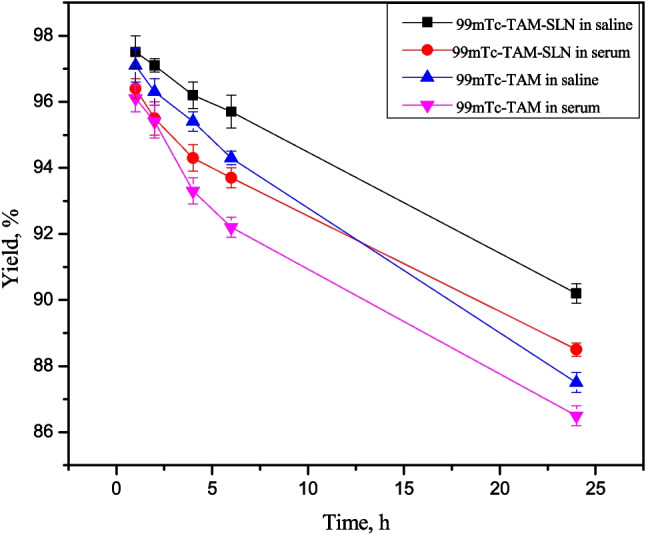
In-vitro stability of [^99m^Tc]Tc-TAM-SLNs and [^99m^Tc]Tc-TAM solution

The evaluation of serum stability is essential throughout the first stage of the drug development process. The labelled substance may degrade or change as a result of serum enzymes, resulting in lower performance in vivo. So before doing the biodistribution investigation, the stability of [^99m^Tc]Tc-TAM-SLNs and [^99m^Tc]Tc-TAM solution at body temperature (37 °C) with serum and saline were evaluated, and they were stable up to 6 h, as cleared from Fig. 4.

### In vivo biodistribution of the radiolabelled compounds in different animal groups

Table 3 showed the in vivo biodistribution of [^99m^Tc]Tc-TAM and [^99m^Tc]Tc-TAM-SLNs in normal Swiss Albino mice after injection. Both [^99m^Tc]Tc-TAM solution and [^99m^Tc]Tc-TAM-SLNs were rapidly distributed in different body organs. High liver and kidney uptake may be explained as the medication was metabolized in the liver and mainly removed by the kidneys [40]. Table 4 showed the uptake of both [^99m^Tc]-TAM solution and [^99m^Tc]Tc-TAM-SLNs in solid tumor-bearing mice, the % accumulation in body organs and fluids expressed as % injected dose/g organ (% ID/g organ ± SD for three mice per group). The blood uptake of [^99m^Tc]Tc-TAM solution was 11.5% at 15 min post injection, while that of [^99m^Tc]Tc-TAM-SLNs was 13.7% and decreased to 1.7% and 1.5% at 240 min post injection. A significant decrease in both [^99m^Tc]Tc-TAM solution and [^99m^Tc]Tc-TAM-SLNs uptake in the majority was observed at 2 and 4 h post injection. Following four hours of dosing, most of the labelled compounds were removed from the systemic circulation. The result in Table 4 can also indicate that the nanoparticles can accumulate in a tumor via the enhanced permeation retention (EPR) effect or nanomaterials-induced endothelial leakiness. Moreover, a high concentration with prolonged retention of the nanoparticles can provide more drug release to tumor cells and potentially induce more cell death (ID% 6.6 ± 0.3* after 4 h).

**Table 3:** Biodistribution of [^99m^Tc]Tc-TAM and [^99m^Tc]Tc-TAM-SLNs in normal mice

Organs & body fluids		% ID/gram tissue at different time intervals			
		1/4 h	1 h	2 h	4 h
Blood	[^99m^Tc]Tc-TAM	12.7 ± 1.1	10.2 ± 0.7*	5.1 ± 0.4*	2.5 ± 0.2*
	**[**^**99m**^**Tc]Tc-TAM-SLNs**	**14.7 ± 1.2**	11.3 ± 0.6*	5.9 ± 0.4*	2.7 ± 0.2*
Bone	[^99m^Tc]Tc-TAM	0.5 ± 0.01	0.7 ± 0.01*	0.9 ± 0.01*	0.6 ± 0.01*
	**[**^**9 m**^**Tc]Tc-TAM-SLNs**	**0.7 ± 0.01**	1.3 ± 0.02*	0.8 ± 0.01*	**0.7 ± 0.01**
Muscle	[^99m^Tc]Tc-TAM	0.9 ± 0.01	1.5 ± 0.03*	1.7 ± 0.08	1.4 ± 0.07*
	**[**^**99m**^**Tc]Tc-TAM-SLNs**	**0.7 ± 0.01**	1.2 ± 0.03*	1.1 ± 0.02*	1.4 ± 0.01*
Liver	[^99m^Tc]Tc-TAM	5.3 ± 0.4	7.8 ± 0.5*	8.9 ± 0.6	4.9 ± 0.3*
	**[**^**99m**^**Tc]Tc-TAM-SLNs**	**8.3 ± 0.5**	11.8 ± 0.8*	9.9 ± 0.6*	3.9 ± 0.2*
Lung	[^99m^Tc]Tc-TAM	2.2 ± 0.1	3.9 ± 0.2*	3.6 ± 0.2	2.5 ± 0.2*
	**[**^**99m**^**Tc]Tc-TAM-SLNs**	**2.7 ± 0.2**	4.8 ± 0.3*	3.9 ± 0.2*	2.9 ± 0.2*
Heart	[^99m^Tc]Tc-TAM	5.5 ± 0.4	4.5 ± 0.3*	3.7 ± 0.2*	2.1 ± 0.1*
	**[**^**99m**^**Tc]Tc-TAM-SLNs**	**6.2 ± 0.4**	3.5 ± 0.2*	2.2 ± 0.1*	1.2 ± 0.01*
Stomach	[^99m^Tc]Tc-TAM	3.8 ± 0.2	5.5 ± 0.3*	6.3 ± 0.4	3.3 ± 0.2
	**[**^**99m**^**Tc]Tc-TAM-SLNs**	**4.8 ± 0.3**	6.5 ± 0.4*	3.3 ± 0.2*	2.3 ± 0.1*
Intestine	[^99m^Tc]Tc-TAM	2.4 ± 0.2	4.4 ± 0.3*	5.1 ± 0.4	3.5 ± 0.2*
	**[**^**99m**^**Tc]Tc-TAM-SLNs**	**4.4 ± 0.3**	**5.3 ± 0.3**	7.1 ± 0.5*	4.5 ± 0.3*
Kidney (urine)	[^99m^Tc]Tc-TAM	5.9 ± 0.4	13.4 ± 0.9*	16.2 ± 1.1*	23.8 ± 1.2*
	**[**^**99m**^**Tc]Tc-TAM-SLNs**	**7.9 ± 0.5**	12.2 ± 0.8*	**14.2 ± 1.2**	21.8 ± 1.3*
Spleen	[^99m^Tc]Tc-TAM	1.1 ± 0.07	1.6 ± 0.1*	0.9 ± 0.04*	0.7 ± 0.03*
	**[**^**99m**^**Tc]Tc-TAM-SLNs**	**0.9 ± 0.04**	1.4 ± 0.08*	0.8 ± 0.04*	0.5 ± 0.02*

Values represent mean ± SD. n = 3

^*^Means significantly differ from the previous each value using unpaired student’s t-test p < 0.05)

**Table 4:** Biodistribution of [^99m^Tc]Tc-TAM and [^99m^Tc]Tc-TAM-SLNs in solid tumor bearing mice

Organs & body fluids		% ID/gram tissue at different time intervals			
		15 min	1 h	2 h	4 h
Blood	[^99m^Tc]Tc-TAM	11.5 ± 0.9	8.4 ± 0.5*	4.8 ± 0.03*	1.7 ± 0.09*
	**[**^**99m**^**Tc]Tc-TAM-SLNs**	**13.7 ± 1.1**	9.3 ± 0.6*	5.4 ± 0.3*	1.5 ± 0.07*
Bone	[^99m^Tc]Tc-TAM	0.7 ± 0.04	1.3 ± 0.07*	1.1 ± 0.06	1.0 ± 0.05
	**[**^**99m**^**Tc]Tc-TAM-SLNs**	**0.8 ± 0.03**	1.4 ± 0.07*	0.9 ± 0.05*	1.1 ± 0.05*
Muscle	[^99m^Tc]Tc-TAM	1.1 ± 0.05	1.2 ± 0.06	1.3 ± 0.07	1.1 ± 0.05
	**[**^**99m**^**Tc]Tc-TAM-SLNs**	**0.9 ± 0.06**	**1.0 ± 0.05**	**1.1 ± 0.06**	0.8 ± 0.04*
Liver	[^99m^Tc]Tc-TAM	4.6 ± 0.3	7.7 ± 0.5*	6.3 ± 0.4*	5.1 ± 0.3*
	**[**^**99m**^**Tc]Tc-TAM-SLNs**	**6.3 ± 0.4**	8.7 ± 0.5*	6.9 ± 0.4*	3.8 ± 0.2*
Lung	[^99 m^Tc]Tc-TAM	2.9 ± 0.2	3.8 ± 0.2*	3.3 ± 0.2	2.1 ± 0.1*
	**[**^**99m**^**Tc]Tc-TAM-SLNs**	**2.1 ± 0.1**	3.9 ± 0.2*	3.1 ± 0.2*	2.4 ± 0.1*
Heart	[^99m^Tc]Tc-TAM	5.4 ± 0.3	4.5 ± 0.3*	2.9 ± 0.2*	1.8 ± 0.1*
	**[**^**99m**^**Tc]Tc-TAM-SLNs**	**6.6 ± 0.4**	5.1 ± 0.3*	3.5 ± 0.2*	2.8 ± 0.2*
Stomach	[^99m^Tc]Tc-TAM	3.2 ± 0.2	5.2 ± 0.3*	4.1 ± 0.3*	2.1 ± 0.1*
	**[**^**99m**^**Tc]Tc-TAM-SLNs**	**4.5 ± 0.3**	6.3 ± 0.4*	3.3 ± 0.2*	2.2 ± 0.1*
Intestine	[^99m^Tc]Tc-TAM	4.1 ± 0.3	4.4 ± 0.3	4.9 ± 0.3*	3.1 ± 0.2*
	**[**^**99m**^**Tc]Tc-TAM-SLNs**	**5.3 ± 0.3**	6.4 ± 0.4*	**5.1 ± 0.3**	3.5 ± 0.2*
Kidney (urine)	[^99m^Tc]Tc-TAM	8.9 ± 0.5	11.4 ± 0.6*	14.2 ± 1.0*	19.8 ± 1.2*
	**[**^**99m**^**Tc]Tc-TAM-SLNs**	**7.9 ± 0.4**	13.4 ± 0.8*	16.2 ± 1.1*	12.8 ± 1.0*
Spleen	[^99m^Tc]Tc-TAM	1.4 ± 0.09	1.9 ± 0.1*	1.1 ± 0.05*	0.8 ± 0.04*
	**[**^**99m**^**Tc]Tc-TAM-SLNs**	**1.2 ± 0.07**	1.5 ± 0.09*	**1.3 ± 0.08**	1.1 ± 0.06*
Tumor muscle	[^99m^Tc]Tc-TAM	1.2 ± 0.06	1.7 ± 0.09*	2.1 ± 0.1*	2.4 ± 0.2
	**[**^**99m**^**Tc]Tc-TAM-SLNs**	**3.2 ± 0.2**	5.6 ± 0.4*	7.2 ± 0.5*	6.6 ± 0.3*

Values represent mean ± SD. n = 3

^*^Means significantly differ from the previous each value using unpaired student’s t-test p < 0.05)

### (T/NT) ratio

**Fig. 5:**
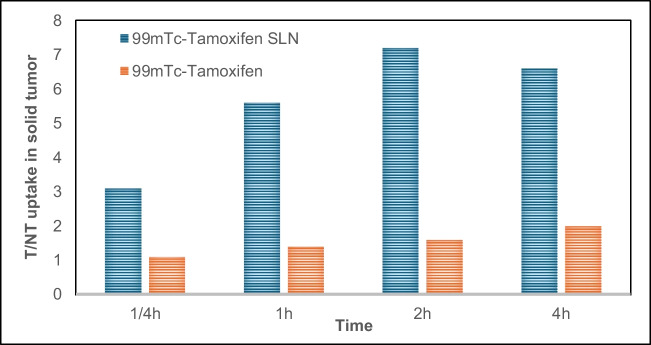
Comparison between [^99m^Tc]Tc-TAM-SLNs and [^99m^Tc]Tc-TAM uptake in solid tumor

The targets to non-target ratios (T/NT) ratio of [^99m^Tc]Tc-TAM-SLNs were 5.6, 7.2, and 6.6 at 1, 2, and 4 h, suggesting significant affinity of the [^99m^Tc]Tc-TAM-SLNs complex to the tumor tissue. As seen in Fig. 5, the data demonstrated that the target to non-target ratio (T/NT) in solid tumor cells for [^99m^Tc]Tc-TAM-SLNs was approximately 3.4 times higher than that generated from [^99m^Tc]Tc-TAM solution. According to the findings, [^99m^Tc]Tc-TAM-SLNs may be used as a model delivery system to administer chemotherapy medications to cancerous cells.

### Pharmacokinetic parameters

The C_max_, T_max_, AUC_(0–240)_, AUC_(0—∞)_, DTE and RTE calculated pharmacokinetic parameters of both [^99m^Tc]Tc-TAM solution and [^99m^Tc]Tc-TAM-SLNs were displayed in Table 5. The C_max_ of the [^99m^Tc]Tc-TAM-SLNs was 7.9 µg/ml at 240 min (T_max_), while that [^99m^Tc]Tc-TAM solution was 2.4 µg/ml at 120 min (T_max_) after I.V. administration. Moreover, the AUC _(0–240)_ and the AUC _(0–∞)_ of the [^99m^Tc]Tc-TAM-SLNs were significantly higher than that of the plain drug (7.63 and 23.45 min %ID/g and 12.43 and 34.05), respectively (p < 0.05). IV administration of SLNs was reported to have numerous pros, such as a large surface area, high stability, controlled drug release, non-toxicity, and release at specific sites where they accumulate [41].

**Table 5:** The Pharmacokinetic parameters of [^99 m^Tc]Tc-TAM and [^99 m^Tc]Tc-TAM-SLNs

Pharmacokinetic parameter	[^99 m^Tc]Tc-TAM	[^99 m^Tc]Tc-TAM-SLNs
**C**_**max**_** (%/g)**	**2.4**	7.9
T_max_ (min)	240	120
AUC_0—240_ (min%ID/g)	7.63	23.45
AUC_0—∞_ (min%ID/g)	12.43	34.05
DTE	0.34	0.94
RTE		3.07

Impressively, the %DTE revealed almost no noticeable variation in the distribution of radiolabelled TAM and radiolabelled encapsulated TAM across different organs in normal mice, whereas in solid tumor-bearing mice the %DTE of the [^99m^Tc]Tc-TAM-SLNs was about 3 times compared to that of the [^99m^Tc]Tc-TAM. The explanation of these results is due to abnormalities and defective vasculature in cancer angiogenesis that facilitate the easy penetration of SLNs at nano-sized lipid particles, leading to enhanced SLN uptake and storage in the tumor [42, 43]. Moreover, well-designed nanoparticle technology, like SLNs, can be used to target tumors passively by utilizing the enhanced permeability and retention (EPR) effect. Low venous return and lymphatic drainage can contribute to the prolonged maintenance of increased SLNs concentrations in the tumor [44]. This approach offers a partial solution to the poor tissue specificity problems and the dangerous consequent side effects [45].

## Conclusion

Our study demonstrated the successful preparation of TAM-SLNPs at the nanoscale that marks a major breakthrough in targeted cancer therapy. With a polydispersity index (PDI) of 0.601 ± 0.3 and an average particle size of 202.5 ± 0.3 nm, these nanoparticles show encouraging properties that enhance their stability and effectiveness. Notably their physiological stability of six hours suggests a suitable timeframe for therapeutic application, and their radiolabelling purity of 97.2 ± 2.4% highlights the possibility of precise imaging and tracking within the body. Most importantly, the high ability of [^99m^Tc]Tc-TAM-SLNs to target cancerous areas makes them an extremely powerful cancer-fighting tool and this might result in better treatment outcomes and fewer side effects for patients receiving breast cancer treatments. These findings supported that TAM-SLNs at the nanoscale might be used as a unique medicinal delivery mechanism to specifically target tumors and also [^99m^Tc]Tc-TAM-SLNs could be considered as a recommended radiopharmaceutical model for tumor imaging.

## Data Availability

The datasets generated during and/or analyzed during the current study are available from the corresponding author on reasonable request.
